# Comment on “A Better Way to Decrease Knee Swelling in Patients with Knee Osteoarthritis: A Single-Blind Randomised Controlled Trial”

**DOI:** 10.1155/2019/2619437

**Published:** 2019-09-29

**Authors:** Max Broere, Yoshka van der Tuijn, Ingrid A. Szilagyi, Erin M. Macri

**Affiliations:** Department of General Practice, Erasmus MC University Medical Center, Rotterdam, Netherlands

With great interest, we read the article of Sari et al. [1] about decreasing knee swelling in patients with knee osteoarthritis using intermittent pneumatic compression (IPC). To our knowledge, this is the first study describing the use of IPC as a treatment for knee osteoarthritis. The authors found significant effects on reducing knee swelling. The topic of research is relevant as knee osteoarthritis is the most common chronic joint disorder affecting millions of people worldwide [2]; however, after reading the article, several questions regarding the study remain unanswered to us. We hope the authors can provide us more insight into this study.

Firstly, the title, introduction, results, and discussion suggest that the authors' main focus in this study was knee swelling. In the first statement of the introduction, knee swelling is described as the most common symptom of knee osteoarthritis; however, the literature generally describes that knee OA typically involves knee pain, stiffness, and loss of function. While swelling is not uncommon, we respectfully believe this statement may be overstating the clinical importance of swelling in knee OA. To complicate matters, in the sample size calculation (Section 2.5 in the article), knee joint range of motion (ROM) was reported to be the primary outcome measure—this does not match the stated aims of the study. To try and clarify this discrepancy, we looked up the study protocol, which stated that knee swelling and pain intensity were the primary outcome measures. Unfortunately, we also noticed that the trial protocol was registered several years after completion of data collection (the actual study completion date is June 1, 2012) and just 5 days before the manuscript was submitted for publication. We cannot help but wonder if ROM was in fact once the primary outcome, but this cannot be confirmed given the very late registration of the trial protocol. The purpose of a trial protocol is to ensure research integrity. We would like to recommend avoiding the inappropriate use of the protocol registration system for future research.

Secondly, some details about participant characteristics and dropouts have not been reported in this manuscript. We noticed that all 9 participants who were lost to follow-up were in the cold pack group (CP group). It remains unclear when participants were informed about which group they were allocated to, so could these 9 dropouts in one group be related to a lack of allocation concealment? In addition, was this accounted for in the final analyses? If so, how? We respectfully suggest it would be more appropriate to handle dropouts using an intention-to-treat analysis (ITT). According to the CONSORT guidelines on reporting of RCTs [3], the number of participants in each group should be analyzed using an ITT analysis. Furthermore, although the exclusion criteria are well described, the inclusion criteria remain unclear to us. Which definition of the American College of Rheumatology criteria (ACR criteria) [4] did the authors use to include patients? Additionally, the severity of the osteoarthritis and which knee compartments were involved would be interesting to know.

Finally, we respectfully question the use of measuring tape to quantify knee joint swelling and the interpretation of these results. Most outcome measures used in this article are widely accepted and validated, such as the Western Ontario and McMaster Universities Osteoarthritis Index (WOMAC), dynamometer, goniometer, and Visual Analogue Scale (VAS). But the validation of the measuring tape remains unclear. We recognize that the authors acknowledged that imaging would have been a better option but that this was cost-prohibitive and, admittedly, we cannot offer an alternative, equally affordable clinical measure for quantifying swelling objectively. Can the authors comment on this outcome measure's reliability and have properties such as minimal detectable difference or minimal clinically important difference [5] been established in this population? Knowing these values would make it easier to interpret the results. We note the authors have relied solely on *p* values to determine whether treatment was effective. Current best practices encourage authors to report effect sizes and uncertainty metrics in addition to *p* values [6]. Since the authors found no between-group differences in pain or physical function scores, we question whether reduced knee swelling in these patients translates into clinically meaningful improvement. Overall, we encourage the authors to exercise caution in interpreting the results of this study so as not to overstate the results of their work.

In conclusion, we would like to thank the authors for their work. We appreciate the substantial undertaking of conducting a randomized controlled trial and commend the authors for seeking novel treatments to address this important clinical population. We hope our feedback and subsequent author response will serve to improve the reader's interpretation of the current study findings and similar research in the future.
